# Insights Into Human Development and Disease From Human Pluripotent Stem Cell Derived Intestinal Organoids

**DOI:** 10.3389/fmed.2019.00297

**Published:** 2019-12-17

**Authors:** Abdelkader Daoud, Jorge O. Múnera

**Affiliations:** Department of Regenerative Medicine and Cell Biology, Medical University of South Carolina, Charleston, SC, United States

**Keywords:** human pluripotent stem cells, organoids, endoderm, midgut, hindgut

## Abstract

In recent years, advances in human pluripotent stem cell (hPSC) biology have enabled the generation of gastrointestinal (GI) organoids which recapitulate aspects of normal organ development. HPSC derived gastrointestinal organoids are comprised of epithelium and mesenchyme and have a remarkable ability to self-organize and recapitulate early stages of human intestinal development. Furthermore, hPSC derived organoids can be transplanted into immunocompromised mice which allows further maturation of both the epithelium and mesenchyme. In this review, we will briefly summarize work from model systems which has elucidated mechanisms of GI patterning and how these insights have been used to guide the differentiation of hPSCs into organoids resembling small intestine and colon. We will succinctly discuss how developmental principles have been used to promote maturation of human intestinal organoids (HIOs) *in vitro* as well as to introduce an enteric nervous system into HIOs. We will then concisely review how organoids have been used to study human pathogens, how new genetic and bioengineering tools are being applied to organoid research, and how this integration has allowed researchers to elucidate mechanisms of human development and disease. Finally, we will briefly discuss remaining challenges in the field and how they can be addressed. HPSC derived organoids are promising new model systems which hold the potential of unlocking unknown mechanisms of human gastrointestinal development and disease.

## Generation of Intestinal Organoids

In the past decade, organoid models have revolutionized the study of the gastrointestinal (GI) epithelium (1–4). Organoid models have provided significant insights into homeostasis and pathogenesis of GI organs. The most commonly used organoid models are derived from patient tissue, are grown as strictly epithelial structures and it has been suggested that they should be referred to as enteroids and colonoids (5). In addition, since they are derived from patients, they will reflect the age and physiology of the patient. Furthermore, enteroids and colonoids will reflect the region of intestine from which they are derived (4, 6). In contrast, human pluripotent stem cells (embryonic stem cells or induced pluripotent stem cells) can be differentiated into human intestinal and colonic organoids which contain both epithelial and mesenchymal cell types and reflect early stages of human gut development (7, 8).

**Figure 1:**
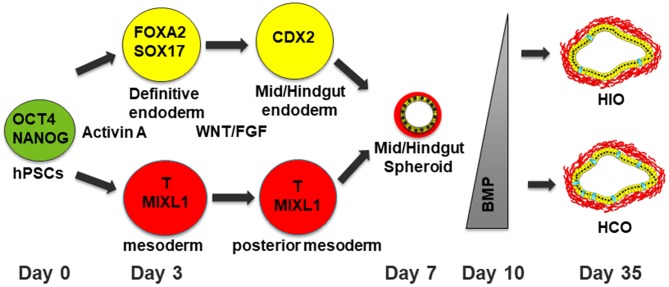
Schematic of differentiation into human intestinal and colonic organoids. Human pluripotent stem cells are differentiated into definitive endoderm (DE) with co-developing mesoderm by treatment with Activin A for 3 days. The resulting DE is then treated with WNT and FGF4 for patterning into mid/hindgut endoderm with posterior mesoderm. After 4 days of treatment with WNT/FGF4, floating spheroids will be evident. These floating spheroids can be plated in a Matrigel bubble and then BMP signaling can either be inhibited for 3 days to generate HIOs or activated for 3 days to generate HCOs with increasing numbers of goblet cells present in HCOs. Human pluripotent stem cells are depicted in green. Definitive endoderm and DE derived cells are depicted in yellow. Mesoderm and mesoderm derived cells are depicted in red. Goblet cells within HIOs and HCOs are depicted in cyan.

Studies in model organisms such as frog, mouse and chick are the foundation for directing human pluripotent stem cells toward desired tissue fates (9). Human intestinal organoids are generated by a stepwise differentiation which involves a series of growth factor manipulations which mimic mammalian development (Figure 1). First, definitive endoderm (DE) is specified using the NODAL mimetic Activin A (10–12). DE induction is not a uniform process so co-developing mesodermal cells are also present in DE monolayers. This developing endoderm/mesoderm can then be patterned into mid/hindgut tissue through activation of the WNT and fibroblast growth factor (FGF) pathways (8). In addition to patterning the endoderm into caudal type homeobox 2 (CDX2) positive mid/hindgut tissue, WNT and FGF also induce 3-dimensional (3D) morphogenesis of endoderm and co-developing mesoderm resulting in the formation of mid/hindgut spheroids which bud off from the endoderm monolayer. These spheroids can then be grown in 3D culture using conditions developed in the Clevers lab (4, 13). This allows the development of human intestinal organoids (HIOs) which undergo morphological transitions similar to what occurs in normal mammalian development. HIOs also generate all the main epithelial and mesenchymal cells types present in the human intestine.

The generation of HIOs from hPSCs can be difficult to reproduce without proper training and experience. Suboptimal seeding density of hPSCs can result in the generation of definitive endoderm incapable of undergoing 3D morphogenesis into mid/hindgut spheroids. To circumvent this, several groups have generated HIOs by simply passaging WNT and FGF treated DE monolayers as clumps (14–17). When grown in 3D culture, these clumps can develop into HIOs which resemble HIOs generated from mid/hindgut spheroids. These studies indicate that patterned DE retains the capacity to self-organize even in the absence of spheroid morphogenesis. In addition, these studies provide an easier alternative to generating HIOs from spheroids.

HIOs have been utilized to study the effects of commensal bacteria on HIO physiology. Hill et al. utilized HIOs to investigate changes in the gene expression within immature human intestinal tissue upon the introduction of commensal *Escherichia coli* (*E. coli*) (18). Bacterial injection into the HIO lumen led to transcriptional changes that are normally associated with innate immune responses, and the activation of the NF-kB pathway. Using a fiber-optic optode to measure luminal oxygen levels in HIOs, the authors demonstrated that the transcriptional changes in HIOs injected with commensal bacteria were partially attributed to microbe-induced hypoxia. As a result, the naïve tissue exhibited an enhanced antibacterial response including increased mucus secretion, increased expression of beta defensins and elevated expression of tight junction proteins following injection of bacteria. This study indicates that commensal microbes can induce a complex physiological response in immature human intestinal tissue similar to responses seen in germ free mice that are colonized with commensal microbes.

## Introducing Complexity and Maturation of HIOs

The co-development of mesoderm within HIOs suggested that there is a signaling crosstalk between the epithelium and the mesenchyme. Subsequent studies demonstrated that HIOs could be grown in the absence of NOGGIN and R-spondin indicating that the HIO mesenchyme is sufficient to maintain organoid growth in the absence of these and other factors, similar to what occurs *in vivo* (19, 20). This led researchers to hypothesize that co-developing mesoderm could support growth of HIOs following transplantation into the kidney capsule of immunocompromised mice. Following transplantation, HIOs matured into tissue that closely resembles human intestine with crypts, villi and all the major epithelial cell types present in the human intestine (19). In addition, the mesoderm from organoids formed circular and longitudinal smooth muscle layers. Engrafted tissue had a functional epithelial barrier, expressed functional brush border enzymes, had functional peptide absorption and had increased crypt fission following ileocecal resection (ICR) of the host. These studies highlight the utility of HIOs to model human gut physiology *in vivo*.

The smooth muscle layers of the intestine are innervated by the enteric nervous system which allows for peristalsis. The development of the smooth muscle layers in transplanted HIOs suggested that HIOs could be innervated (19). The enteric nervous system develops from vagal neural crest cells (NCCs). In order to establish an enteric nervous system in intestinal organoids, HIOs were mixed with vagal NCCs which were also derived from human pluripotent stem cells (21). *In vitro*, NCCs co-cultured with HIOs differentiated into distinct cell types of the human enteric nervous system (ENS) including neurons and glial cells. NCC differentiation and organization into ENS was supported by the HIOs which expressed the chemoattractant factors glial cell derived neurotrophic factor (GDNF) and endothelin 3 (EDN3). The resulting neurons were solely excitatory and displayed a spontaneous calcium efflux indicating a neuronal activity. The complexity of the neuronal system was further enhanced after *in vivo* transplantation resulting in the emergence of inhibitory neurons in addition to the excitatory neurons.

To functionally test the ability of HIOs with an ENS to produce peristalsis, electrical stimulation was applied to transplanted HIOs *ex vivo*. Upon receiving a neuronal stimulus, HIOs with an ENS exhibited contractile waves similar to peristalsis in human intestines suggesting a neuromuscular communication. Additionally, the NCC derived inhibitory neurons integrated into the smooth muscle layers to control tissue relaxation by releasing the neurotransmitter nitric oxide (NO). Furthermore, the presence of ENS following transplantation of the HIOs resulted in decreased expression of markers of differentiated epithelial cell types and increased epithelial proliferation in the HIOs. The generation of innervated intestinal tissue is a significant advancement since it could allow for the replacement of damaged intestine with a tissue which is competent to induce peristalsis.

HIOs grown *in vitro* lack maturity (they lack brush border enzymes, hormone expressing enteroendocrine cells, mature intestinal stem cells, and mature Paneth cells) and thus have limited applications for studying intestinal physiology (21, 22). To address this shortcoming, Jung et al. hypothesized that the co-culture of HIOs with T lymphocytes (Jurkat cells) would allow the *in vitro* maturation of these organoids (23). Surprisingly, this co-culture system allowed the expression of maturation markers such as intestinal stem cell expressed olfactomedin 4 (OLFM4), Paneth cell expressed defensin alpha 5 (DEFA5), brush border expressed sucrase isomaltase (SI), dipeptidyl Peptidase 4 DPP4, lactase (LCT), and enteroendocrine cell expressed gastric inhibitory polypeptide (GIP) in HIOs grown *in vitro*. The authors were able to identify Interleukin 2 (IL-2) as the crucial factor secreted by Jurkat cells which allowed the maturation of the HIOs *in vitro*. The transcriptomic profile of co-cultured and IL-2 treated HIOs more closely resembled the human adult small intestine. Functional maturation was also evident in the matured HIOs as assessed by their ability to establish barrier function, transport chemotherapeutic drugs and transport glucose. Additionally, matured HIOs expressed functional cystic fibrosis transmembrane conductance regulator (CFTR) and were capable of swelling in response to forskolin, an assay designed to screen for Cystic Fibrosis therapeutic drugs (24, 25). The group postulated that IL-2-induced maturation of HIOs could substitute the *in vivo* maturation and provide an opportunity for large-scale organoid generation, gene editing and drug screening. Although these results were intriguing, it should be noted that HIOs are able to mature following renal transplantation into NOD scid gamma (NSG) mice which lack lymphocytes and have normal intestinal development.

## Regional Patterning of HIOs

Initial analysis of HIOs revealed that they were comprised of proximal and distal small intestinal tissue even within the same organoid suggesting additional signaling might be required for proper patterning of HIOs (8). In order to pattern organoids into region specific segments, two different groups took different approaches. The first group extended the mid-hindgut induction step to show that prolonged WNT and FGF4 signaling could promote the development of distal small intestine (26). This work shed new light on the significance of the dose and length of exposure to growth factors on intestinal regionalization. While short activation of WNT and FGF4 patterned the intestinal organoids into a developing duodenum following renal transplantation, prolonged stimulation led to the development of an intestinal tissue with decreased PDX1, GATA4, ONECUT2, and DMBT1 expression and higher SATB2, GUACA2A, MUC2, and FABP6 levels suggesting an ileal identity.

In another study, Múnera et al. demonstrated that a transient activation of BMP signaling was sufficient to induce the expression of posterior Homeobox (HOX) genes and confer a colonic identity on human gut tube cultures (7). Furthermore, the authors demonstrated that patterning was induced in both the epithelium and mesenchyme of human colonic organoids (HCOs). The resulting HCOs expressed colon enriched HOX genes and special AT-Rich sequence-binding protein 2 (SATB2), a chromatin modifying nuclear protein. In addition, following prolonged *in vitro* culture, HCOs generated colon enriched cell types such as mucin 5B (MUC5B) expressing goblet cells and the colon specific enteroendocrine hormone insulin like 5 (INSL5) following induced expression of NEUROG3. After renal transplantation into immunocompromised mice, HCOs retained the expression of SATB2, lacked GATA4 expression and lacked small intestinal cell types such as GIP expressing enteroendocrine cells and Paneth cells. In contrast, transplanted HIOs expressed GATA4 and contained small intestinal cell types such as Paneth cells and small intestinal enteroendocrine hormones such as Ghrelin, Motilin, and GIP. These studies demonstrated the utility of hPSC-derived organoids for studying mechanisms of anterior-posterior patterning.

## Modeling of Infectious Disease Using HIOs

Enteric viruses such as rotaviruses and noroviruses are the main infectious agents which cause gastroenteritis and are a primary cause of infant mortality in developing countries (27). Despite their global disease burden, rotaviruses and noroviruses have been difficult to study because suitable *in vitro* models for viral replication are scarce. HIOs have offered some hope as they have been shown to allow limited replication of clinical isolates of rotaviruses and noroviruses (22, 28). The limited replication of these viruses may be due to the fetal nature of HIOs and efforts to improve the *in vitro* maturation may directly benefit research on enteric viruses. Thus, organoid technology may offer a unique opportunity for future development of vaccines and therapeutics agents for enteric viruses.

**Figure 2:**
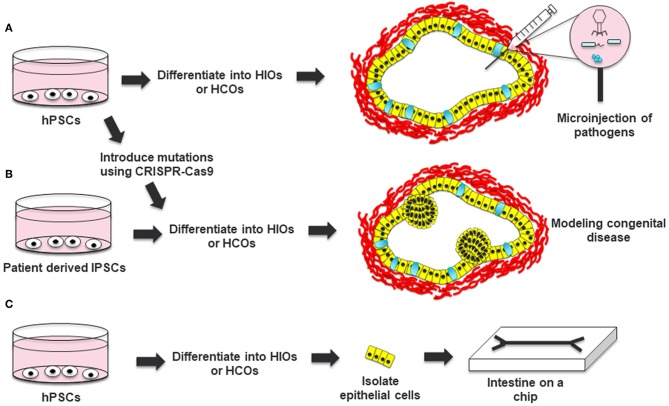
Applications of HIOs and HCOs. **(A)** Human pluripotent stem cells can be differentiated into HIOs or HCOs and microinjected with bacterial or viruses to study host-pathogen interaction. **(B)** Induced pluripotent stem cells from patients with a congenital disease or hPSCs which are edited to carry a pathogenic mutation can be differentiated into HIOs and used to model the disease. **(C)** Epithelial cells from generated HIOs can be isolated and then placed in a microchip to test the effects of luminal flow and luminal contents on epithelial physiology. Definitive endoderm derived epithelia are depicted in yellow. Mesoderm derived cells are depicted in red. Goblet cells within HCOs are depicted in cyan.

HIOs have also been extensively used to study bacterial pathogens including *Salmonella enterica* Serovar Typhimurium, Shiga toxin (Stx) producing *Escherichia coli* O157:H7, and *Clostridium difficile*. Bacterial pathogens can be microinjected into the lumen of HIOs to study the effect of the pathogens on the epithelium and mesenchyme of HIOs. These studies demonstrated that HIOs recapitulate responses to bacterial pathogens including internalization of bacteria, increased secretion of mucus, epithelial barrier disruption and induced pro-inflammatory cytokine expression (29–33). Lees et al. also showed that IL22 can protect the epithelium of HIOs from *Salmonella* infection (17). In addition, Karve et al. demonstrated that cytokines expressed by HIOs could recruit co-cultured neutrophils into the infected lumen of the organoid (29). This suggests that HIOs combined with immune cells could be an attractive model for studying interactions between pathogens, epithelia and immune cells (Figure 2A).

## Genetic Tools in HIO Research

HIOs can be used as a tool for studying gene function in the intestine (Figure 2B). In the original work by Spence et al., the authors used adenovirus and lentivirus to study the gain-of-function and loss-of-function of NEUROG3, respectively (8). Adenovirus mediated overexpression of NEUROG3 resulted in an increased number of enteroendocrine cells in HIOs while knockdown of NEUROG3 using lentivirus expressing short hairpin RNAs (shRNAs) resulted in a decreased number of enteroendocrine cells (EECs). Expanding on this work, McCracken an co-workers developed a NEUROG3-inducible system to promote the generation of EECs in gastrointestinal organoids (34). This system has been instrumental to studying the expression of regionally expressed enteroendocrine hormones (7, 34, 35). In addition, this system has been used to interrogate the functionality of EECs, including the regulation of satiety and hunger inducing hormones in response to luminal nutrients (36).

CRISPR-Cas9 gene editing has also been applied to hPSCs and hPSC-derived HIOs. NEUROG3 deficient hPSCs were previously developed to interrogate the role of NEUROG3 in the development of pancreatic endocrine progenitors (37). Capitalizing on this NEUROG3 null system and the NEUROG3-inducible system, Zhang et al. utilized these tools to study the physiology of mutant forms of NEUROG3 on the development of endocrine cells in the pancreas and intestine (38). The authors were able to systematically interrogate the biochemical and functional properties of six different NEUROG3 mutations including those related to protein stability, dimerization and DNA binding. CRISPR-Cas9 gene editing has also been used to study genes associated with Hirschsprung's disease, a disease in which the large intestine is not properly innervated (21). By introducing a mutation in PHOX2B, researchers were able to demonstrate that PHOX2B deficient NCCs were impaired in their development into cells of the ENS and this resulted in impaired smooth muscle differentiation and *in vivo* growth of HIOs. CDX2 deficient hPSCs have also been generated and these cells were used to demonstrate the role of this transcription factor in the specification of mid/hindgut vs. foregut fate (39).

The ability to reprogram adult somatic cells into induced pluripotent stem cells (iPSCs) allows the generation of patient specific iPSCs (40). Using this reprogramming approach, Woo et al. generated iPSCs from patients with Dyskeratosis Congenita (DC), a disease which results in shortened telomeres leading to stem cell exhaustion in highly proliferative tissues (41). By using CRISPR-Cas9 gene editing they were able to correct the genetic defect in patient derived cells. Additionally, they demonstrated that WNT signaling was suboptimal in HIOs derived from DC patient iPSCs and that this could be compensated by the addition of a WNT agonist which resulted in increased telomerase activity, telomere length and telomere capping. Additionally, two different groups generated iPSCs from patients with Familial adenomatous polyposis (FAP) (16, 42). Both groups were able to demonstrate that organoids grown from FAP patient-derived iPSCs exhibited increased WNT signaling compared to controls. To minimize genetic background effects, the group from Boston University used Talen mediated gene editing to correct the mutant copy of the Adenomatous Polyposis Coli (APC) gene to examine differences between FAP and FAP-corrected HIOs. In contrast, the group from Cornell used normal donors as controls but adjusted organoid growth conditions to generate HCOs. They then used FAP patient derived HCOs to screen for drugs which could restore the expression of APC protein to levels seen in wild-type HCOs. Taken together, these studies demonstrate the utility of HIOs and HCOs for modeling human congenital diseases and for drug screening.

## Bioengineering and HIOs

The generation of HIOs can be an inefficient process which could limit their scalability. To address this shortcoming, Arora et al. investigated the impact of spheroid number, size and morphology on organoid yield and found the last two parameters to be the key features that help identify spheroids which are most likely to generate organoids (43). By developing an image assisted automated micropipette aspiration and release system, the team was able to isolate “pre-organoids” which led to a significant increase in organoid formation. This study highlights how using a bioengineering approach to select spheroids for plating can increase organoid yields.

The use of Matrigel as a substrate for HIO growth raises concerns since Matrigel contains the non-human sialic acid Neu5Gc which humans have antibodies against. Thus, alternatives to Matrigel will be required for transplantation of organoids in humans. Synthetic gels could provide a replacement to animal derived extracellular matrices. Cruz-Acuna et al. used a synthetic poly-ethylene glycol-based hydrogel to demonstrate that hydrogels could support the growth of HIOs *in vitro*. In addition, the authors were able to demonstrate that HIOs grown in this hydrogel could be orthotopically transplanted and could improve mucosal wound healing in the colon of immunocompromised mice (44). A similar study by Capeling et al. demonstrated that Alginate gels could also support the growth and differentiation of HIOs *in vitro* and *in vivo* following renal transplantation into immunocompromised mice (45). These studies have provided proof-of-concept that synthetic matrices can be used for HIO growth and transplantation.

HIOs form an internal lumen which hinders the ability to study the effects of bacteria or other factors on the luminal surface of the epithelium. Although protocols have been developed that allow growth of human enteroids and colonoids as monolayers allowing luminal access, these protocols have not been adapted for HIOs (46, 47). To address the problem of luminal access in HIOs, Workman et al. isolated epithelial cells from HIOs and grew them in microchips (Figure 2C) (48). This allowed the researchers to optimize the speed of luminal flow necessary for the proper polarization and differentiation of the intestinal epithelial cells. The researchers were able to use these intestinal cells on a microchip to study epithelial barrier function in response to luminally administered cytokines. Platforms like this should allow for high throughput analysis of barrier function in response to luminal signals and also help integrate other cell types present in the intestine such as immune cells.

Transplanted HIOs grow as spherical structures with a closed lumen even following transplantation into immunocompromised mice. Developing tissue for transplantation will require the development of tube-like organoids that can be engrafted and that they will maintain luminal flow. In order to generate more tube-like intestinal tissue from HIOs, Poling et al. implanted uniaxial springs into the lumens of HIOs to examine the effect of mechanical stretching on HIO physiology (49). Besides allowing the generation of tube-like HIOs, the mechanical forces applied by the spring allowed an enhanced *in vivo* maturation of transplanted HIOs based on morphometric analysis, global transcriptional analysis, and functional assays. Moreover, the device increased the tone of the smooth muscles generated by the HIOs. This study highlights how mechanical force can improve organoid morphology and maturation and should aid attempts to transplant such tissue.

## Future Directions

HIOs hold tremendous promise for studying the role of epithelial, mesenchymal and neuronal cell types in intestinal physiology and disease. However, immune cells are still absent in HIOs cultures. Multiple immune cell types are present in human intestine including various macrophage subtypes, dendritic cells and lymphocytes (50). The use of developmentally inspired methods for generating and co-culturing immune cells should allow for unprecedented studies examining the interaction of immune cells with the various cell types that are present in HIOs. This will allow for improved models of complex diseases such as inflammatory bowel disease (IBD) and intestinal cancers. It should also allow for the interrogation of the developmental origins of gut associated lymphoid tissues (GALT).

The use of microinjection to study bacterial interaction with the epithelium of HIOs is laborious and low-throughput. Recently, Williamson et al. developed a high-throughput platform to microinject biopsy derived organoids (also referred to as colonoids) (51). This platform also allows for automated imaging through visualization of organoids over time. Adapting this platform for use with HIOs and HCOs should allow high-throughput analysis of bacterial pathogens and microbiota.

Although morphologically HIOs undergo transitions similar to those that occur in mammalian embryos, the molecular changes associated with these transitions remain unresolved. Future experiments directly comparing HIOs to human fetal and postnatal intestine from several stages of development will determine the relative age of an HIO at any timepoint during the *in vitro* culture. In addition, single cell RNA sequencing of HIOs compared to fetal intestine should allow the identification of cell types which are differentially absent or present in HIOs compared to human fetal intestine.

## Author Contributions

AD and JM helped to write and edit the manuscript.

### Conflict of Interest

The authors declare that the research was conducted in the absence of any commercial or financial relationships that could be construed as a potential conflict of interest.

## References

[1] Date S Sato T. Mini-gut organoids: reconstitution of the stem cell niche. Annu Rev Cell Dev Biol. (2015) 31:269–89. 10.1146/annurev-cellbio-100814-125218 26436704

[2] Fujii M Clevers H Sato T. Modeling human digestive diseases with CRISPR-Cas9-modified organoids. Gastroenterology. (2019) 156:562–76. 10.1053/j.gastro.2018.11.048 30476497

[3] Matano M Date S Shimokawa M Takano A Fujii M Ohta Y. Modeling colorectal cancer using CRISPR-Cas9-mediated engineering of human intestinal organoids. Nat Med. (2015) 21:256–62. 10.1038/nm.3802 25706875

[4] Sato T Stange DE Ferrante M Vries RG Van Es JH Van den Brink S. Long-term expansion of epithelial organoids from human colon, adenoma, adenocarcinoma, and Barrett's epithelium. Gastroenterology. (2011) 141:1762–72. 10.1053/j.gastro.2011.07.050 21889923

[5] Stelzner M Helmrath M Dunn JC Henning SJ Houchen CW Kuo C. A nomenclature for intestinal *in vitro* cultures. Am J Physiol Gastrointest Liver Physiol. (2012) 302:G1359–63. 10.1152/ajpgi.00493.2011 22461030 PMC3378093

[6] Middendorp S Schneeberger K Wiegerinck CL Mokry M Akkerman RD van Wijngaarden S. Adult stem cells in the small intestine are intrinsically programmed with their location-specific function. Stem Cells. (2014) 32:1083–91. 10.1002/stem.1655 24496776

[7] Múnera JO Sundaram N Rankin SA Hill D Watson C Mahe M. Differentiation of human pluripotent stem cells into colonic organoids via transient activation of BMP signaling. Cell Stem Cell. (2017) 21:51–64.e6. 10.1016/j.stem.2017.05.020 28648364 PMC5531599

[8] Spence JR Mayhew CN Rankin SA Kuhar MF Vallance JE Tolle K. Directed differentiation of human pluripotent stem cells into intestinal tissue *in vitro*. Nature. (2011) 470:105–9. 10.1038/nature09691 21151107 PMC3033971

[9] Zorn AM Wells JM. Vertebrate endoderm development and organ formation. Annu Rev Cell Dev Biol. (2009) 25:221–51. 10.1146/annurev.cellbio.042308.113344 19575677 PMC2861293

[10] D'Amour KA Agulnick AD Eliazer S Kelly OG Kroon E Baetge EE. Efficient differentiation of human embryonic stem cells to definitive endoderm. Nat Biotechnol. (2005) 23:1534–41. 10.1038/nbt1163 16258519

[11] Lowe LA Yamada S Kuehn MR. Genetic dissection of nodal function in patterning the mouse embryo. Development. (2001) 128:1831–43. 11311163 10.1242/dev.128.10.1831

[12] Conlon FL Lyons KM Takaesu N Barth KS Kispert A Herrmann B. A primary requirement for nodal in the formation and maintenance of the primitive streak in the mouse. Development. (1994) 120:1919–28. 7924997 10.1242/dev.120.7.1919

[13] Sato T Vries RG Snippert HJ van de Wetering M Barker N Stange DE. Single Lgr5 stem cells build crypt-villus structures *in vitro* without a mesenchymal niche. Nature. (2009) 459:262–5. 10.1038/nature07935 19329995

[14] Fordham RP Yui S Hannan NR Soendergaard C Madgwick A Schweiger PJ. Transplantation of expanded fetal intestinal progenitors contributes to colon regeneration after injury. Cell Stem Cell. (2013) 13:734–44. 10.1016/j.stem.2013.09.015 24139758 PMC3858813

[15] Tamminen K Balboa D Toivonen S Pakarinen MP Wiener Z Alitalo K. Intestinal commitment and maturation of human pluripotent stem cells is independent of exogenous FGF4 and R-spondin1. PLoS ONE. (2015) 10:e0134551. 10.1371/journal.pone.0134551 26230325 PMC4521699

[16] Crespo M Vilar E Tsai SY Chang K Amin S Srinivasan T. Colonic organoids derived from human induced pluripotent stem cells for modeling colorectal cancer and drug testing. Nat Med. (2017) 23:878–84. 10.1038/nm.4355 28628110 PMC6055224

[17] Lees EA Forbester JL Forrest S Kane L Goulding D Dougan G Using human induced pluripotent stem cell-derived intestinal organoids to study and modify epithelial cell protection against salmonella and other pathogens. J Vis Exp. (2019) 147:e59478 10.3791/59478 31132035

[18] Hill DR Huang S Nagy MS Yadagiri VK Fields C Mukherjee D. Bacterial colonization stimulates a complex physiological response in the immature human intestinal epithelium. Elife. (2017) 6:e29132. 10.7554/eLife.29132.031 29110754 PMC5711377

[19] Watson CL Mahe MM Munera J Howell JC Sundaram N Poling HM. An *in vivo* model of human small intestine using pluripotent stem cells. Nat Med. (2014) 20:1310–4. 10.1038/nm.3737 25326803 PMC4408376

[20] Farin HF Van Es JH Clevers H. Redundant sources of Wnt regulate intestinal stem cells and promote formation of Paneth cells. Gastroenterology. (2012) 143:1518–29.e7. 10.1053/j.gastro.2012.08.031 22922422

[21] Workman MJ Mahe MM Trisno S Poling HM Watson CL Sundaram N. Engineered human pluripotent-stem-cell-derived intestinal tissues with a functional enteric nervous system. Nat Med. (2017) 23:49–59. 10.1038/nm.4233 27869805 PMC5562951

[22] Finkbeiner SR Zeng XL Utama B Atmar RL Shroyer NF Estes MK. Stem cell-derived human intestinal organoids as an infection model for rotaviruses. MBio. (2012) 3:e00159-12. 10.1128/mBio.00159-12 22761392 PMC3398537

[23] Jung KB Lee H Son YS Lee MO Kim YD Oh SJ. Interleukin-2 induces the *in vitro* maturation of human pluripotent stem cell-derived intestinal organoids. Nat Commun. (2018) 9:3039. 10.1038/s41467-018-05450-8 30072687 PMC6072745

[24] Dekkers JF Wiegerinck CL de Jonge HR Bronsveld I Janssens HM de Winter-de Groot KM. A functional CFTR assay using primary cystic fibrosis intestinal organoids. Nat Med. (2013) 19:939–45. 10.1038/nm.3201 23727931

[25] Schwank G Koo BK Sasselli V Dekkers JF Heo I Demircan T. Functional repair of CFTR by CRISPR/Cas9 in intestinal stem cell organoids of cystic fibrosis patients. Cell Stem Cell. (2013) 13:653–8. 10.1016/j.stem.2013.11.002 24315439

[26] Tsai YH Nattiv R Dedhia PH Nagy MS Chin AM Thomson M. *In vitro* patterning of pluripotent stem cell-derived intestine recapitulates *in vivo* human development. Development. (2017) 144:1045–55. 10.1242/dev.138453 27927684 PMC5358103

[27] Girard MP Steele D Chaignat CL Kieny MP. A review of vaccine research and development: human enteric infections. Vaccine. (2006) 24:2732–50. 10.1016/j.vaccine.2005.10.014 16483695

[28] Zhang D Tan M Zhong W Xia M Huang P Jiang X. Human intestinal organoids express histo-blood group antigens, bind norovirus VLPs, and support limited norovirus replication. Sci Rep. (2017) 7:12621. 10.1038/s41598-017-12736-2 28974702 PMC5626734

[29] Karve SS Pradhan S Ward DV Weiss AA. Intestinal organoids model human responses to infection by commensal and Shiga toxin producing *Escherichia coli*. PLoS ONE. (2017) 12:e0178966. 10.1371/journal.pone.0178966 28614372 PMC5470682

[30] Forbester JL Goulding D Vallier L Hannan N Hale C Pickard D. Interaction of *Salmonella enterica* Serovar Typhimurium with intestinal organoids derived from human induced pluripotent stem cells. Infect Immun. (2015) 83:2926–34. 10.1128/IAI.00161-15 25964470 PMC4468523

[31] Leslie JL Huang S Opp JS Nagy MS Kobayashi M Young VB. Persistence and toxin production by Clostridium difficile within human intestinal organoids result in disruption of epithelial paracellular barrier function. Infect Immun. (2015) 83:138–45. 10.1128/IAI.02561-14 25312952 PMC4288864

[32] Engevik MA Engevik KA Yacyshyn MB Wang J Hassett DJ Darien B. Human Clostridium difficile infection: inhibition of NHE3 and microbiota profile. Am J Physiol Gastrointest Liver Physiol. (2015) 308:G497–509. 10.1152/ajpgi.00090.2014 25552580 PMC4422371

[33] Engevik MA Yacyshyn MB Engevik KA Wang J Darien B Hassett DJ. Human Clostridium difficile infection: altered mucus production and composition. Am J Physiol Gastrointest Liver Physiol. (2015) 308:G510–24. 10.1152/ajpgi.00091.2014 25552581 PMC4422372

[34] McCracken KW Cata EM Crawford CM Sinagoga KL Schumacher M Rockich BE. Modelling human development and disease in pluripotent stem-cell-derived gastric organoids. Nature. (2014) 516:400–4. 10.1038/nature13863 25363776 PMC4270898

[35] McCracken KW Aihara E Martin B Crawford CM Broda T Treguier J. Wnt/beta-catenin promotes gastric fundus specification in mice and humans. Nature. (2017) 541:182–7. 10.1038/nature21021 28052057 PMC5526592

[36] Sinagoga KL McCauley HA Munera JO Reynolds NA Enriquez JR Watson C. Deriving functional human enteroendocrine cells from pluripotent stem cells. Development. (2018) 145:dev165795. 10.1242/dev.165795 30143540 PMC6198470

[37] McGrath PS Watson CL Ingram C Helmrath MA Wells JM. The basic helix-loop-helix transcription factor NEUROG3 is required for development of the human endocrine pancreas. Diabetes. (2015) 64:2497–505. 10.2337/db14-1412 25650326 PMC4477351

[38] Zhang X McGrath PS Salomone J Rahal M McCauley HA Schweitzer J. A comprehensive structure-function study of neurogenin3 disease-causing alleles during human pancreas and intestinal organoid development. Dev Cell. (2019) 50:367–80.e7. 10.1016/j.devcel.2019.05.017 31178402 PMC7082840

[39] Kumar N Tsai YH Chen L Zhou A Banerjee KK Saxena M. The lineage-specific transcription factor CDX2 navigates dynamic chromatin to control distinct stages of intestine development. Development. (2019) 146:dev172189. 10.1242/dev.172189 30745430 PMC6432663

[40] Takahashi K Tanabe K Ohnuki M Narita M Ichisaka T Tomoda K. Induction of pluripotent stem cells from adult human fibroblasts by defined factors. Cell. (2007) 131:861–72. 10.1016/j.cell.2007.11.019 18035408

[41] Woo DH Chen Q Yang TL Glineburg MR Hoge C Leu NA. Enhancing a Wnt-telomere feedback loop restores intestinal stem cell function in a human organotypic model of Dyskeratosis Congenita. Cell Stem Cell. (2016) 19:397–405. 10.1016/j.stem.2016.05.024 27545506 PMC7900823

[42] Sommer CA Capilla A Molina-Estevez FJ Gianotti-Sommer A Skvir N Caballero I. Modeling APC mutagenesis and familial adenomatous polyposis using human iPS cells. PLoS ONE. (2018) 13:e0200657. 10.1371/journal.pone.0200657 30024920 PMC6053155

[43] Arora N Imran Alsous J Guggenheim JW Mak M Munera J Wells JM. A process engineering approach to increase organoid yield. Development. (2017) 144:1128–36. 10.1242/dev.142919 28174251 PMC5358111

[44] Cruz-Acuna R Quiros M Huang S Siuda D Spence JR Nusrat A. PEG-4MAL hydrogels for human organoid generation, culture, and *in vivo* delivery. Nat Protoc. (2018) 13:2102–19. 10.1038/s41596-018-0036-3 30190557 PMC7240347

[45] Capeling MM Czerwinski M Huang S Tsai YH Wu A Nagy MS. Nonadhesive alginate hydrogels support growth of pluripotent stem cell-derived intestinal organoids. Stem Cell Rep. (2019) 12:381–94. 10.1016/j.stemcr.2018.12.001 30612954 PMC6373433

[46] In J Foulke-Abel J Zachos NC Hansen AM Kaper JB Bernstein HD. Enterohemorrhagic *Escherichia coli* reduce mucus and intermicrovillar bridges in human stem cell-derived colonoids. Cell Mol Gastroenterol Hepatol. (2016) 2:48–62.e3. 10.1016/j.jcmgh.2015.10.001 26855967 PMC4740923

[47] VanDussen KL Marinshaw JM Shaikh N Miyoshi H Moon C Tarr PI. Development of an enhanced human gastrointestinal epithelial culture system to facilitate patient-based assays. Gut. (2015) 64:911–20. 10.1136/gutjnl-2013-306651 25007816 PMC4305344

[48] Workman MJ Gleeson JP Troisi EJ Estrada HQ Kerns SJ Hinojosa CD. Enhanced utilization of induced pluripotent stem cell-derived human intestinal organoids using microengineered chips. Cell Mol Gastroenterol Hepatol. (2018) 5:669–77.e2. 10.1016/j.jcmgh.2017.12.008 29930984 PMC6009013

[49] Poling HM Wu D Brown N Baker M Hausfeld TA Huynh N. Mechanically induced development and maturation of human intestinal organoids *in vivo*. Nat Biomed Eng. (2018) 2:429–42. 10.1038/s41551-018-0243-9 30151330 PMC6108544

[50] Mowat AM Agace WW. Regional specialization within the intestinal immune system. Nat Rev Immunol. (2014) 14:667–85. 10.1038/nri3738 25234148

[51] Williamson IA Arnold JW Samsa LA Gaynor L DiSalvo M Cocchiaro JL. A high-throughput organoid microinjection platform to study gastrointestinal microbiota and luminal physiology. Cell Mol Gastroenterol Hepatol. (2018) 6:301–19. 10.1016/j.jcmgh.2018.05.004 30123820 PMC6092482

